# Reassessment of American Joint Committee on Cancer Staging for Stage III Renal Cell Carcinoma With Nodal Involvement: Propensity Score Matched Analyses of a Large Population-Based Study

**DOI:** 10.3389/fonc.2020.00365

**Published:** 2020-03-19

**Authors:** Jianglong Han, Qin Li, Ping Li, Shijie Wang, Rui Zhang, Yunfeng Qiao, Qibin Song, Zhenming Fu

**Affiliations:** Cancer Center, Renmin Hospital of Wuhan University, Wuhan, China

**Keywords:** renal cell carcinoma, lymph node metastases, SEER, staging, nomogram, propensity score matching

## Abstract

**Background:** To assess the role of nodal involvement in stage III renal cell carcinoma (RCC) according to the American Joint Committee on Cancer (AJCC) 8th staging system. We compared the survival outcomes of RCC patients with pT_1−3_N_1_M_0_ disease and those with pT_3_N_0_M_0_ or stage IV (stratified as pT_4_N_any_M_0_ and pT_any_N_any_M_1_) disease in a large population-based cohort.

**Methods:** A cohort of 3,112 eligible patients with RCC was identified from the Surveillance, Epidemiology, and End Results (SEER) database, registered between January 2004 and December 2015. Kaplan-Meier and Cox proportional hazards models were used to evaluate the overall survival (OS), and cancer-specific survival (CSS). The prognostic value of the modified stage for pT_1−3_N_1_M_0_ disease was assessed by nomogram-based analyses. Propensity score matching (PSM) was used to adjust for potential baseline confounding.

**Results:** Patients with pT_1−3_N_1_M_0_ disease showed similar survival outcomes (median OS 41.0 vs. 38.0 months, *P* = 0.77; CSS 45.0 vs. 39.0 months, *P* = 0.59) to pT_4_N_any_M_0_ patients, whereas the significantly better survival outcome was found for pT_3_N_0_M_0_ patients. After PSM, comparable survival rates were observed between pT_1−3_N_1_M_0_ group and pT_4_N_any_M_0_ group, which were still significantly worse than the survival of pT_3_N_0_M_0_ patients. The modified stage IIIA (pT_3_N_0_M_0_), IIIB (pT_1−3_N_1_M_0_, pT_4_N_any_M_0_), and IV (pT_any_N_any_M_1_) showed higher predictive accuracy than AJCC stage system in the nomogram-based analyses (concordance index: 0.70 vs. 0.68, *P* < 0.001 for OS; 0.71 vs. 0.69, *P* < 0.001 for CSS).

**Conclusions:** The pT_1−3_N_1_M_0_ RCC might be reclassified as stage IIIB together with pT_4_N_any_M_0_ disease for better prediction of prognosis, further examination and validation are warranted.

## Introduction

Renal cell carcinoma (RCC) ranks the third most common genitourinary malignancy in men and fourth among women, with an estimated 403,262 new cases and 175,098 deaths worldwide (1). Lymph node (LN) involvement accounts for 6% to 20% in patients diagnosed with RCC (2, 3). The 5-years overall survival (OS) was significantly worse in node-positive patients ranging from 11 to 38% compared to 65 to 87% relative to those without nodal disease (2, 4, 5). Positive node disease has been frequently shown to have an independent adverse effect on survival, regardless of other prognostic factors (6, 7). However, even though the determinant prognostic role of LN involvement might exist in the survival of RCC patients, the current 8th version of the American Joint Committee on Cancer (AJCC) staging manual classifies both the pT_1−3_N_1_M_0_ disease and pT_3_N_0_M_0_ disease as stage III disease (8).

Several studies suggested that RCC patients with pT_1−3_N_1_M_0_ disease were associated with poor survival compared with RCC patients with pT_3_N_0_M_0_ disease (9, 10). Furthermore, the survival between RCC patients with M_1_ disease (N_0_M_1_) and patients with node-positive only (N_+_M_0_) disease was similar (11). The recent MD Anderson Cancer Center (MDACC) study reported that RCC patients with pT_1−3_N_1_M_0_ disease by the AJCC 8th staging should be reclassified as having stage IV disease (12). Meanwhile, another large cohort from China also suggested that T_1−3_N_1_M_0_ disease should be reclassified to be combined with T_4_N_0_M_0_ rather than T_3_N_0_M_0_ disease (13).

Therefore, given the heterogeneity of survival outcomes using the current AJCC staging system, we sought to analyze the RCC cohorts of patients from the Surveillance, Epidemiology, and End Results (SEER) registry to improve stratification of survival outcomes, including pT_3_N_0_M_0_, pT_1−3_N_1_M_0_, pT_4_N_any_M_0_, and pT_any_N_any_M_1_ patient populations.

## Methods

### Study Populations and Data Sources

**Figure 1:**
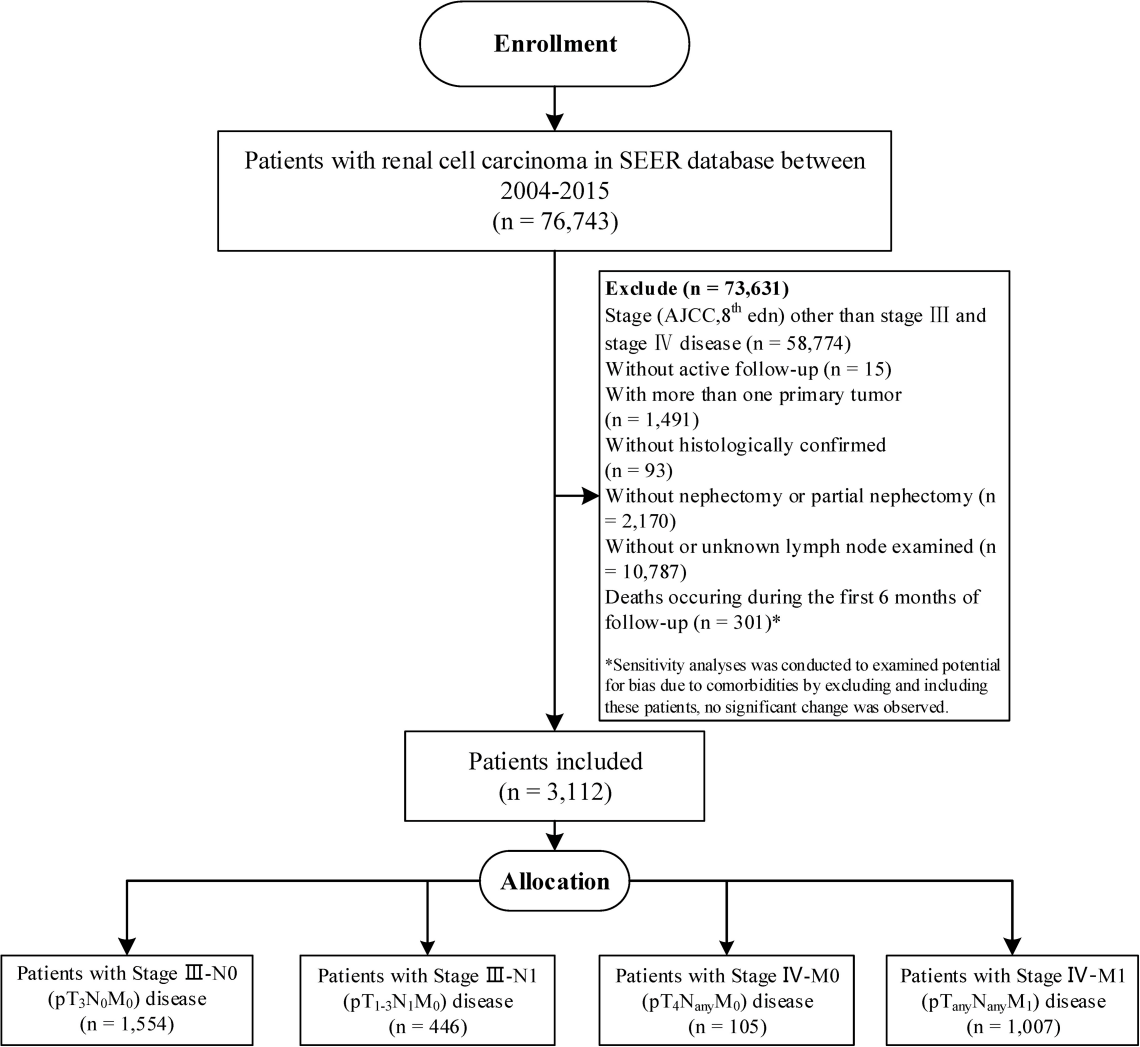
The flowchart of study population selection.

Patient consent was not required because the study was a retrospective database research in nature, there was no direct patient contact. Institutional Review Board approval was not required according to our institution policy. The SEER program of the National Cancer Institute is an authoritative source on cancer incidence and survival in the US covering approximately 34.6% of the US population, which routinely collects basic demographics and some clinical characteristics (14). SEER^*^Stat software (version 8.3.5) was queried to identify patients from SEER-18 database if they were diagnosed with RCC (International Classification of Disease for Oncology, 3rd edition [ICD-O-3], C67.0–C67.9) between January 2004 and December 2015 (*n* = 76,743). The study cohort was then limited to patients with stage III disease or pT_any_N_any_M_1_ and pT_4_N_any_M_0_ disease according to the AJCC 8th Staging Manual using the collaborative stage information (*n* = 17,969). The eligible criteria included in the study were participants with one primary only, pathologically confirmed RCC, and contained complete data of age, race, gender, surgery records, pathological information, and with active follow up; and all patients underwent radical or partial nephrectomy with LN dissection which guaranteed the specimen and harvested LNs were sent for pathology in SEER (15). As a result, the study included a total of 3,112 patients—1,554 patients with pT_3_N_0_M_0_ disease, 446 patients with pT_1−3_N_1_M_0_ disease, 105 patients with pT_4_N_any_M_0_ disease, and 1,007 patients with pT_any_N_any_M_1_ disease (Figure 1).

### Description of Covariates

Baseline characteristics include age (continuous variable), sex (male, female), race (white, non-white), tumor site (left, right), and histological subtypes (clear cell, papillary, and chromophobe). Grade in SEER is coded according to the ICD-O-3. The number of examined and positive LNs is queried from collaborative stage information and presented as a continuous variable. Since information of invasion beyond capsule, fuhrman nuclear grade, and sarcomatoid features are only applicable after 2010, these covariates are not examined in χ^2^-test (16).

### Propensity Score Matching (PSM)

PSM is a tool to reduce selection bias in observational studies (17). PSM with 1:1 matching was performed in our study to reduce the selection bias and ensure baseline balance among study groups. The covariates selected for matching were based on prior literature reports, known clinical prognostic factors, and statistical differences in the multivariate analysis. The variables were first forced into univariate analysis based on the prior literature reports and clinical significance. Then, variables that remained significant in the univariate analysis entered into multivariate Cox regression models. Variables left in the final multivariate analysis were used to generate the propensity score. Selected variables included age (18, 19) stage (8), grade (20), and histology (21) (Figure S1).

### Statistical Analysis

Overall survival (OS) was defined as the time from diagnosis to death from any cause; cancer-specific survival (CSS) was defined as the time from diagnosis to death related to RCC. Frequencies and proportions, as well as means were reported for categorical and continuous variables, respectively. General linear models or χ^2^-test were performed to compare the distribution of baseline characteristics. The survival curves were estimated by the Kaplan-Meier method and compared by the log-rank test. The univariate and multivariate analyses and hazard ratios (HR) were evaluated by Cox proportional hazards regression model to find its independent prognostic risks. Significant variables in univariate analysis were entered into a multivariate model, and variables that remained significant were further entered into the final multivariate regression model. *P* ≤ 0.05 (2-sided probability) was considered statistically significant.

A nomogram consisted of modified stage based on the multivariate analyses was established using the *rms* packages in R (22). The concordance index (C-index) and calibration curve were performed to assess the discriminatory powers of the nomogram. The larger the C-index was, the more accurate the prognostic prediction demonstrated (23). Comparisons between the nomogram and current AJCC stage were performed with the *survcomp* packages in R (24). The predictive performance of model was further examined in the randomly 1 to 1 ratio selected internal validation cohort (25). Although validated in an internal cohort, the predictive accuracy of the modified staging system might be still suffer from some unmeasured confounding. All analyses were conducted using R software (version 3.5.3). Sensitivity analyses were conducted to investigate the potential for bias due to the existence of comorbidities by including and excluding deaths occurring during the first 6 months of follow-up.

## Results

Between January 2004 and December 2015, 3,112 eligible patients were identified and categorized into pT_3_N_0_M_0_ group (*n* = 1,554), pT_1−3_N_1_M_0_ group (*n* = 446), pT_4_N_any_M_0_ group (*n* = 105), and pT_any_N_any_M_1_ group (*n* = 1,007). Tables 1, 2 summarize the baseline characteristics for study groups before and after matching (for more information, please see Table S1). Before matching, slightly more non-white patients were included in pT_1−3_N_1_M_0_ group (19.5 vs. 12.9%, *P* < 0.001), patients in pT_1−3_N_1_M_0_ group are more likely to have undifferentiated tumors (22.9 vs. 17.7%, *P* < 0.001) and papillary adenocarcinoma (31.2 vs. 4.3%, *P* < 0.001) than those in pT_3_N_0_M_0_ group. After PSM, all variables between the study groups were balanced, except for those variables that were only applicable after 2010.

**Table 1:** Baseline characteristics of pT_3_N_0_M_0_ and pT_1−3_N_1_M_0_ groups before and after matching, SEER 2004-2015.

**Characteristic**	**Before matching**			After matching^*^		
	**Stage III-N0**	**Stage III-N1**	***P^†^***	**Stage III-N0**	**Stage III-N1**	***P^†^***
	**pT_**3**_N_**0**_M_**0**_**	**pT_**1−3**_N_**1**_M_**0**_**		**pT_**3**_N_**0**_M_**0**_**	**pT_**1−3**_N_**1**_M_**0**_**	
	**(*N* = 1554, %)**	**(*N* = 446, %)**		**(*N* = 373, %)**	**(*N* = 373, %)**	
Age, mean (95%CI), y	60.2 (59.7–60.8)	60.2 (59.1–61.3)	0.93	60.1 (59.0–61.3)	60.5 (59.3–61.7)	0.70
Sex			0.085			0.34
Male	1059 (68.1)	323 (72.4)		257 (68.9)	269 (72.1)	
Female	495 (31.9)	123 (27.6)		116 (31.1)	104 (27.9)	
Race			<0.001			0.14
White	1354 (87.1)	359 (80.5)		319 (85.5)	304 (81.5)	
Non-white	200 (12.9)	87 (19.5)		54 (14.5)	69 (18.5)	
Grade			<0.001			1.0
Well/moderately	442 (28.4)	81 (18.2)		74 (19.8)	74 (19.8)	
Poor	705 (45.4)	224 (50.2)		164 (44.0)	164 (44.0)	
Undifferentiated	275 (17.7)	102 (22.9)		96 (25.7)	96 (25.7)	
Unknown	132 (8.5)	39 (8.7)		39 (10.5)	39 (10.5)	
Tumor site			0.56			0.66
Left	885 (56.9)	247 (55.4)		207 (55.5)	201 (53.9)	
Right	669 (43.1)	199 (44.6)		166 (44.5)	172 (46.1)	
Histology			<0.001			1.0
Clear cell	1407 (90.5)	286 (64.1)		286 (76.7)	286 (76.7)	
Chrompohobe	80 (5.1)	21 (4.7)		21 (5.6)	21 (5.6)	
Papillary	67 (4.3)	139 (31.2)		66 (17.7)	66 (17.7)	
T stage			NA			NA
T1	0 (0)	61 (13.6)		0 (0)	48 (12.9)	
T2	0 (0)	91 (18.2)		0 (0)	70 (18.8)	
T3a	934 (60.1)	216 (48.4)		234 (62.7)	176 (47.2)	
T3b	527 (33.9)	71 (15.9)		123 (33.0)	65 (17.4)	
T3c	60 (3.9)	10 (2.2)		13 (3.5)	8 (2.1)	
T3NOS	33 (2.1)	7 (1.6)		3 (0.8)	6 (1.6)	
N stage			NA			NA
N0	1554 (100.0)	0 (0.0)		373 (100.0)	0 (0.0)	
N1	0 (0.0)	446 (100.0)		0 (0.0)	373 (100.0)	
Invasion beyond capsule^‡^			NA			NA
No	426 (38.3)	148 (50.0)		92 (33.0)	121 (50.0)	
Yes	600 (53.9)	122 (41.2)		165 (59.1)	102 (42.1)	
Unknown	87 (7.8)	26 (8.8)		22 (7.9)	19 (7.9)	
Fuhrman nuclear grade^‡^			NA			NA
Grade 1/2	330 (29.6)	37 (12.5)		67 (24.0)	34 (14.0)	
Grade 3	489 (43.9)	143 (48.3)		114 (40.9)	99 (40.9)	
Grade 4	217 (19.5)	84 (28.4)		76 (27.2)	81 (33.5)	
Unknown	77 (6.9)	32 (10.8)		22 (7.9)	28 (11.6)	
Sarcomatoid features^‡^			NA			NA
Absence	1016 (91.3)	248 (83.8)		247 (88.5)	200 (82.6)	
Presence	62 (5.6)	37 (12.5)		25 (9.0)	35 (14.5)	
Unknown	35 (3.1)	11 (3.7)		7 (2.5)	7 (2.9)	
LNs examined, mean (95%CI)	9 (8–10)	9 (7–11)	0.96	10 (8–12)	9 (7–11)	0.30
LNs positive, mean (95%CI)	0 (0–0)	6 (4–8)	NA	0 (0–0)	6 (4–8)	NA

*LN, lymph node; CI, Confidence interval; NA, Not applicable*.

* *Adjusted for group, age, grade, and histology*.

† *Derived from χ^2^-test for categorical variables, general linear models for continuous variables*.

‡ *Invasion beyond capsule, fuhrman nuclear grade, and sarcomatoid features were only applicable for 2010+ cases, and not enter into the χ^2^-test, thus the P-value was not applicable*.

**Table 2:** Baseline characteristics of pT_1−3_N_1_M_0_ and pT_4_N_any_M_0_ groups before and after matching, SEER 2004-2015.

**Characteristic**	**Before matching**			After matching^*^		
	**Stage III-N1**	**Stage IV-M0**	***P^†^***	**Stage III-N1**	**Stage IV-M0**	***P^†^***
	**pT_**1−3**_N_**1**_M_**0**_**	**pT_**4**_N_**any**_M_**0**_**		**pT_**1−3**_N_**1**_M_**0**_**	**pT_**4**_N_**any**_M_**0**_**	
	**(*N* = 446, %)**	**(*N* = 105, %)**		**(*N* = 105, %)**	**(*N* = 105, %)**	
Age, mean (95%CI), y	60.2 (59.1–61.3)	59.7 (57.8–61.6)	0.71	61.5 (59.3–63.6)	59.7 (57.8–61.6)	0.23
Sex			0.034			0.39
Male	323 (72.4)	65 (61.9)		71 (67.6)	65 (61.9)	
Female	123 (27.6)	40 (38.1)		34 (32.4)	40 (38.1)	
Race			0.44			0.56
White	359 (80.5)	88 (83.8)		91 (86.7)	88 (83.9)	
Non-white	87 (19.5)	17 (16.2)		14 (13.3)	17 (16.2)	
Grade			0.25			0.98
Well/moderately	81 (18.2)	13 (12.4)		13 (12.4)	13 (12.4)	
Poor	224 (50.2)	53 (50.5)		53 (50.5)	53 (50.5)	
Undifferentiated	102 (22.9)	32 (30.5)		32 (30.5)	32 (30.5)	
Unknown	39 (8.7)	7 (6.7)		7 (6.7)	7 (6.7)	
Tumor site			0.74			0.58
Left	247 (55.4)	60 (57.1)		64 (61.0)	60 (57.1)	
Right	199 (44.6)	45 (42.9)		41 (39.0)	45 (42.9)	
Histology			0.002			0.99
Clear cell	286 (64.1)	84 (80.0)		84 (80.0)	84 (80.0)	
Chrompohobe	21 (4.7)	6 (5.7)		6 (5.7)	6 (5.7)	
Papillary	139 (31.2)	15 (14.3)		15 (14.3)	15 (14.3)	
T stage			NA			NA
T1	61 (13.6)	0 (0.0)		16 (15.2)	0 (0.0)	
T2	81 (18.2)	0 (0.0)		21 (20.0)	0 (0.0)	
T3a	216 (48.4)	0 (0.0)		48 (45.7)	0 (0.0)	
T3b	71 (15.9)	0 (0.0)		16 (15.2)	0 (0.0)	
T3c	10 (2.2)	0 (0.0)		2 (1.9)	0 (0.0)	
T3NOS	7 (1.6)	0 (0.0)		2 (1.9)	0 (0.0)	
T4	0 (0.0)	105 (100.0)		0 (0.0)	105 (100.0)	
N stage			NA			NA
N0	0 (0.0)	88 (83.8)		0 (0.0)	88 (83.8)	
N1	446 (100.0)	17 (16.2)		105 (100.0)	17 (16.2)	
Invasion beyond capsule^‡^			NA			NA
No	148 (50.0)	12 (17.1)		30 (45.5)	12 (17.1)	
Yes	122 (41.2)	56 (80.0)		30 (45.5)	56 (80.0)	
Unknown	26 (8.8)	2 (1.9)		6 (9.1)	2 (2.9)	
Fuhrman nuclear grade^‡^			NA			NA
Grade 1/2	37 (12.5)	10 (14.3)		8 (12.1)	10 (14.3)	
Grade 3	143 (48.3)	33 (47.1)		25 (37.9)	33 (47.1)	
Grade 4	84 (28.4)	25 (35.7)		29 (43.9)	25 (35.7)	
Unknown	32 (10.8)	2 (2.9)		4 (6.1)	2 (2.9)	
Sarcomatoid features^‡^			NA			NA
Absence	248 (83.8)	55 (78.6)		56 (84.8)	55 (78.6)	
Presence	37 (12.5	14 (20.0)		7 (10.6)	14 (20.0)	
Unknown	11 (3.7)	1 (1.4)		3 (4.5)	1 (1.4)	
LNs examined, mean (95%CI)	9 (7–11)	8 (6–10)	0.70	11 (6–16)	8 (6–10)	0.29
LNs positive, mean (95%CI)	6 (4–8)	1 (0–2)		9 (4–14)	0 (0–0)	

*LN, lymph node; CI, Confidence interval; NA, Not applicable*.

* *Adjusted for group, age, grade, and histology*.

† *Derived from χ^2^-test for categorical variables, general linear models for continuous variables*.

‡ *Invasion beyond capsule, fuhrman nuclear grade, and sarcomatoid features were only applicable for 2010+ cases, and not enter into the χ^2^-test, thus the P-value was not applicable*.

Table 3 summarizes the independent risk factors for survival of stage III RCC patients. Patients with higher tumor grade (*P* < 0.001), higher fuhrman grade (*P* < 0.001), presence of sarcomatoid features (*P* < 0.001), and presence of node disease (*P* < 0.001) were associated with remarkably worse OS and CSS among stage III RCC patients before matching. After matching, similar outcomes were observed for stage III RCC patients (Table S2). Moreover, patients with the chrompohobe type RCC was associated with better OS (*P* = 0.002) and CSS (*P* = 0.001) compared to patients with clear cell type RCC, which indicated the potential survival benefit of chrompohobe type RCC (Figure S2). In the multivariate analysis, pT_1−3_N_1_M_0_ group was found to be associated with poor outcome compared to the pT_3_N_0_M_0_ group both before (HR = 2.5, 95% CI = 2.1–2.9 for OS, HR = 2.6, 95% CI = 2.2–3.2 for CSS) and after matching (HR = 2.5, 95% CI = 1.9–3.1 for OS, HR =2.5, 95% CI = 2.0–3.3 for CSS).

**Table 3:** Univariate and multivariate analyses of overall survival and cause-specific survival to pT_3_N_0_M_0_ and pT_1−3_N_1_M_0_ groups before matching, SEER 2004-2015.

**Characteristic**	**Overall survival**						**Cancer-specific survival**					
	**Univariate**			Multivariate^*^			**Univariate**			Multivariate^*^		
	**HR**	**95% CI**	***P***	**HR**	**95% CI**	***P***	**HR**	**95% CI**	***P***	**HR**	**95% CI**	***P***
Age	1.02	1.01–1.03	<0.001	1.02	1.01–1.03	<0.001	1.01	1.00–1.02	<0.001	1.02	1.01–1.02	<0.001
Sex												
Male (ref.)	1.0	1.0		/	/	/	1.0	1.0		/	/	/
Female	1.1	0.90–1.3	0.48	/	/	/	1.1	0.94–1.3	0.21	/	/	/
Race												
White (ref.)	1.0	1.0		/	/	/	1.0	1.0		/	/	/
Non-white	1.2	0.95–1.4	0.14	/	/	/	1.1	0.88–1.4	0.37	/	/	/
Grade												
Well/moderately (ref.)	1.0	1.0		1.0	1.0		1.0	1.0		1.0	1.0	
Poor	1.7	1.4–2.1	<0.001	1.7	1.4–2.1	<0.001	1.8	1.5–2.3	<0.001	1.7	1.4–2.2	<0.001
Undifferentiated	2.5	2.0–3.1	<0.001	2.4	1.9–3.0	<0.001	2.9	2.2–3.7	<0.001	2.7	2.1–3.5	<0.001
Unknown	1.1	0.75–1.7	0.58	1.2	0.83–1.8	0.31	1.3	0.88–2.0	0.18	1.4	0.94–2.2	0.093
Tumor site												
Left (ref.)	1.0	1.0		/	/	/	1.0	1.0		/	/	/
Right	1.1	0.92–1.3	0.39	/	/	/	1.1	0.93–1.3	0.28	/	/	/
Histology												
Clear cell (ref.)	1.0	1.0		1.0	1.0		1.0	1.0		1.0	1.0	
Chrompohobe	0.40	0.23–0.69	0.001	0.42	0.24–0.73	0.002	0.36	0.19–0.67	0.001	0.36	0.20–0.68	0.001
Papillary	1.8	1.5–2.3	<0.001	1.2	0.92–1.5	0.20	1.9	1.5–2.4	<0.001	1.2	0.93–1.5	0.17
Invasion beyond capsule^†^												
No (ref.)	1.0	1.0		/	/	/	1.0	1.0		/	/	/
Yes	1.1	0.85–1.4	0.55	/	/	/	1.1	0.86–1.4	0.44	/	/	/
Unknown	1.1	0.73–1.7	0.64	/	/	/	1.2	0.79–1.9	0.38	/	/	/
Fuhrman nuclear grade^†^												
Grade 1/2 (ref.)	1.0	1.0		1.0	1.0		1.0	1.0		1.0	1.0	
Grade 3	2.5	1.7–3.6	<0.001	2.2	1.5–3.2	<0.001	2.7	1.8–4.1	<0.001	2.4	1.6–3.6	<0.001
Grade 4	4.5	3.1–6.6	<0.001	3.4	2.3–5.0	<0.001	5.4	3.5–8.2	<0.001	3.9	2.5–6.0	<0.001
Unknown	2.2	1.3–3.8	0.003	1.9	1.1–3.3	0.019	2.8	1.6–4.9	<0.001	2.4	1.4–4.4	0.003
Sarcomatoid features^†^												
Absence (ref.)	1.0	1.0		1.0	1.0		1.0	1.0		1.0	1.0	
Presence	3.0	2.2–4.2	<0.001	1.9	1.3–2.7	0.001	3.2	2.3–4.6	<0.001	1.9	1.3–2.7	0.001
Unknown	0.95	0.51–1.8	0.88	1.1	0.56–2.0	0.87	0.90	0.44–1.8	0.77	0.97	0.50–1.7	0.94
LNs examined	0.99	0.99–1.0	0.28	/	/	/	0.99	0.99–1.0	0.23	/	/	/
LNs positive	1.0	0.99–1.01	0.083	/	/	/.	1.00	1.00–1.01	0.067	/	/	/
Group												
pT_3_N_0_M_0_ (ref.)	1.0	1.0		1.0	1.0		1.0	1.0		1.0	1.0	
pT_1−3_N_1_M_0_	2.5	2.1–2.9	<0.001	2.5	2.1–2.9	<0.001	2.7	2.3–3.2	<0.001	2.6	2.2–3.2	<0.001

*HR, Hazards ratio; CI, Confidence intervals; LN, Lymph node; ref., referent*.

* *Adjusted by age, group, grade, and histology for cases diagnosed between 2004 and 2015*.

† *Invasion beyond capsule, fuhrman nuclear grade, and sarcomatoid features were only applicable for 2010+cases, and adjusted by age, group, histology, and grade for 2010+ cases*.

**Figure 2:**
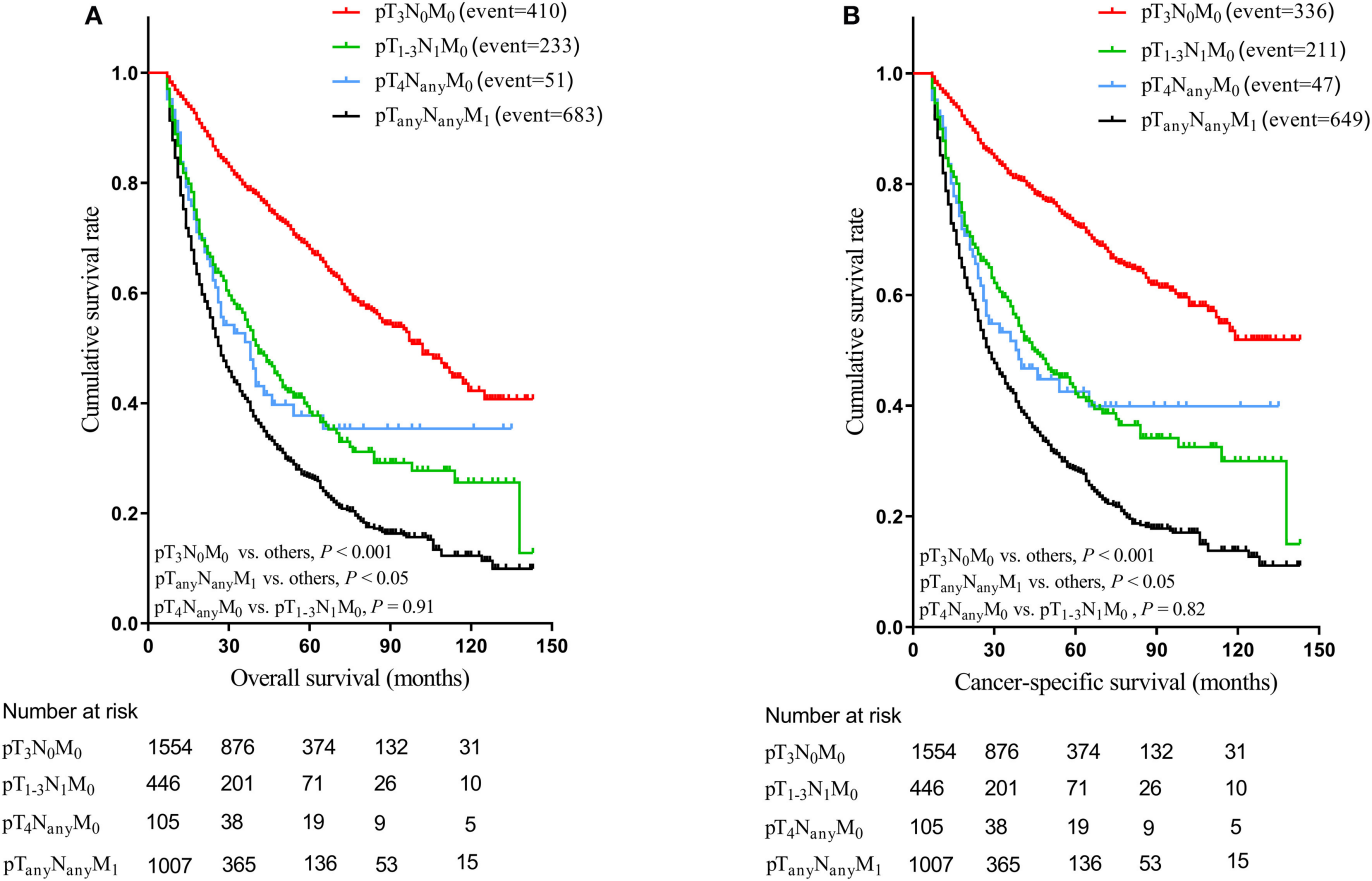
Kaplan-Meier estimation of overall survival curves **(A)** and cancer-specific survival curves **(B)** relative to each group before matching.

**Figure 3:**
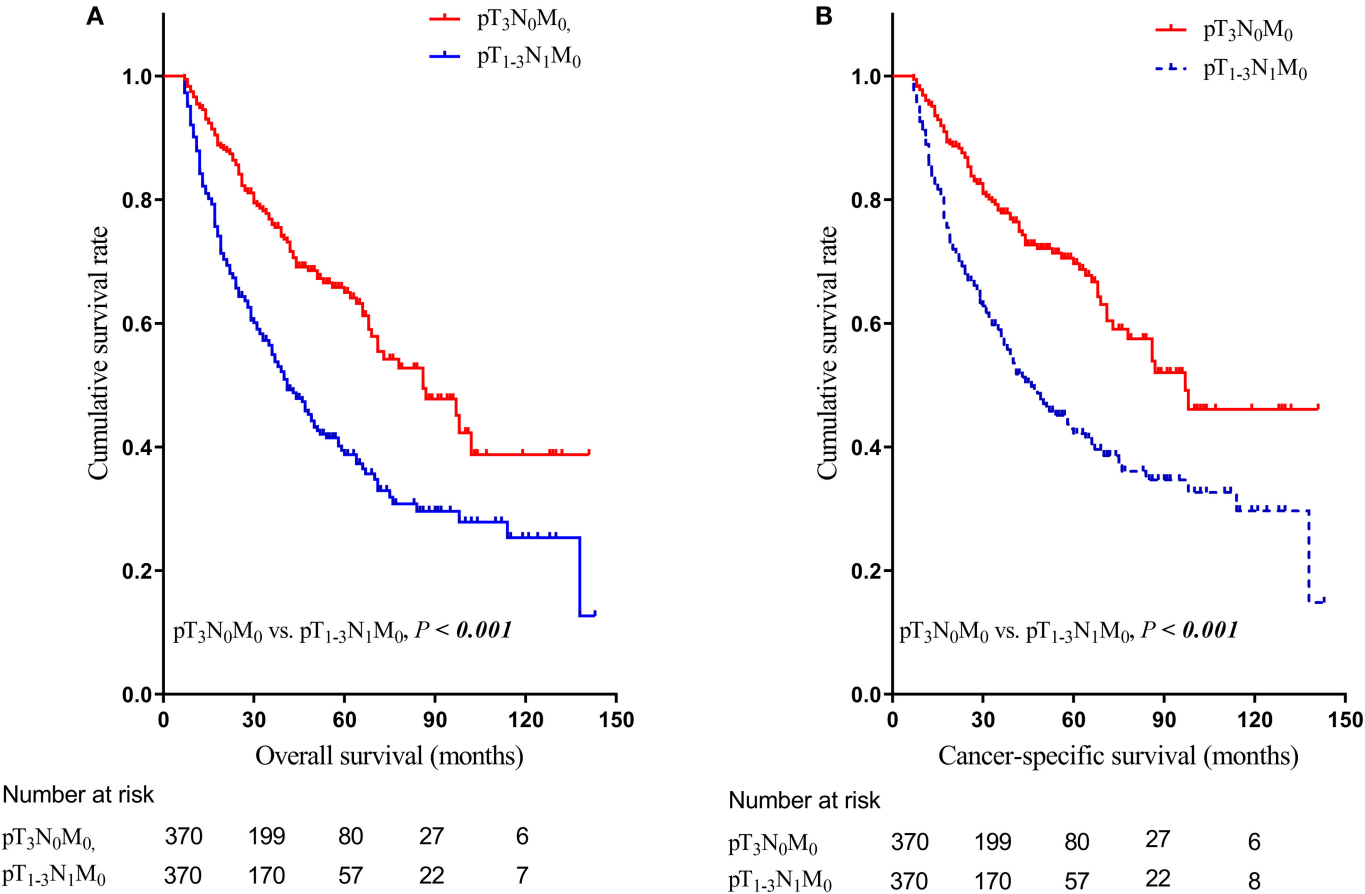
Kaplan-Meier estimation of overall survival curves **(A)** and cancer-specific survival curves **(B)** relative to stage III-N0 (pT_3_N_0_M_0_) and stage III-N1 (pT_1−3_N_1_M_0_) groups after matching.

We further used the Kaplan-Meier curves to demonstrate that the nodal involvement was associated with worse survival. The highest OS and CSS was observed in pT_3_N_0_M_0_ group (median OS = 102.0 months, median CSS = 110.0 months, *P* < 0.001 compared with other groups), whereas the similar survival was found for pT_1−3_N_1_M_0_ group and pT_4_N_any_M_0_ group (*P* = 0.91 for OS, *P* = 0.82 for CSS). Moreover, significantly worse survival (median OS = 27.0 months, median CSS = 28.0 months, *P* < 0.05 compared with other groups) were found for pT_any_N_any_M_1_ group (Figure 2). After matching, significantly better survival was found for pT_3_N_0_M_0_ patients (median OS = 91.0 months, median CSS = 119.0 months, *P* < 0.001) compared with those of pT_1−3_N_1_M_0_ group (Figure 3). In addition, the survival of pT_1−3_N_1_M_0_ group didn't show remarkable difference compared to pT_4_N_any_M_0_ group (Figure S3). However, both pT_1−3_N_1_M_0_ group and pT_4_N_any_M_0_ group were associated with better survival than pT_any_N_any_M_1_ group (Figure S4). Moreover, for those patients diagnosed after 2010, the survival for pT_1−3_N_1_M_0_ group and pT_4_N_any_M_0_ group remained stable both before and after matching (Figure S5).

**Figure 4:**
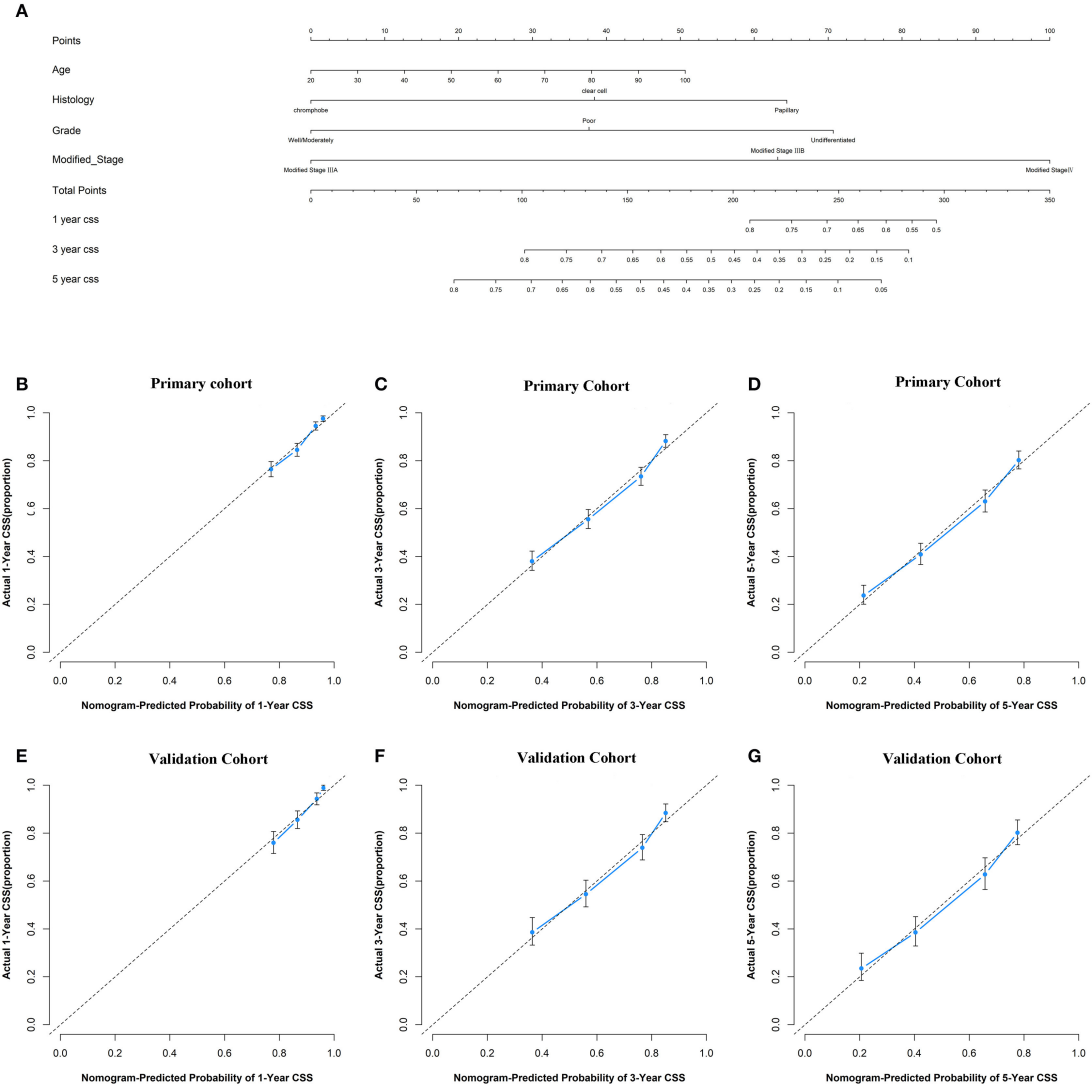
Prognostic nomogram, calibration curves of cancer-specific survival (CSS) for the modified stage of study patients. **(A)** The nomogram predicts 1-, 3-, and 5-years CSS in patients with renal cell carcinoma (RCC). The calibration curves predict CSS at 1-year **(B)**, 3-years **(C)**, and 5-years **(D)** in the primary cohort and at 1-year **(E)**, 3-years **(F)**, and 5-years **(G)** in the validation cohort.

Given the above results, pT_1−3_N_1_M_0_ and pT_4_N_any_M_0_ disease were regrouped into stage IIIB according to the OS and CSS of each subgroup without changing the definition of TNM. pT_3_N_0_M_0_ disease was classified as stage IIIA and pT_any_N_any_M_1_ were classified as stage IV. The prognostic nomogram for modified stage group that integrated significant independent factors for CSS in the primary cohort was shown in Figure 4 (OS shown in Figure S6). The observed probability of 1-, 3-, and 5-years CSS in the primary cohort and 1-, 3-, and 5-years CSS in the validation cohort showed optimal consistency with the nomogram-predicted CSS (Figures 4B–G), the similar results for nomogram-predicted OS were also observed. The C-index for the modified stage group were improved significantly (0.70, 95% CI: 0.68–0.71 vs. 0.68, 95% CI: 0.66–0.69, *P* < 0.001 for OS; 0.71, 95% CI: 0.69–0.73 vs. 0.69, 95% CI: 0.68–0.72, *P* < 0.001 for CSS) compared to the AJCC 8th stage group in the primary cohort as well as the validation cohort (0.71, 95% CI: 0.69–0.73 vs. 0.70, 95% CI: 0.68–0.72, *P* = 0.002 for OS; 0.73, 95% CI: 0.71–0.74 vs. 0.70, 95% CI: 0.69–0.72, *P* < 0.001 for CSS), which indicated the higher discriminatory power of the modified stage.

## Discussion

To the best of our knowledge, the present study is the first population-based analysis using the PSM method to assess the impact of LN involvement on survival in stage III RCC patients. In the current study, LN involvement, in general, was associated with remarkably poor survival in patients with stage III disease in the AJCC 8th staging system in both multivariate regression and PSM analyses. We found that RCC patients with pT_3_N_0_M_0_ disease could be reclassified as stage IIIA, while pT_1−3_N_1_M_0_ RCC together with pT_4_N_any_M_0_ disease could be classified as stage IIIB for better prediction of prognosis.

LN involvement in RCC patients has been decreasing over years, from 30% in historical series to approximately 6%-20% in more recent studies (2, 11, 26–28). Despite the early detection of nodal disease, survival is still poor for those node-positive RCC patients (4, 29, 30). Previous studies consistently showed that node-positive disease was associated with worse survival compared to node-negative disease (4, 6, 7). Capitanio et al. examined the stage-specific effect of nodal involvement in non-metastatic RCC patients based on the 12 institutional databases (4). Of the 2,023 patients enrolled, 165 (4.7%) patients presented with nodal disease. The 5-years CSS rate was 87% for node-negative patients and 38% for node-positive patients (HR = 7.1, *P* < 0.001). Srivastava et al. found the consistent outcomes for node-negative and positive group in their NCDB cohort (median OS, 79.5 vs. 18.6 months) (31). A more recent population-based analysis compared the survival between node-positive disease and node-negative disease patients in the absence of distant metastasis, the 5-years OS rate was 72.7% for pN0 disease patients and 38.1% for pN1 disease patients (*P* < 0.001) (13). Comparable outcomes were observed in our study (5-years OS rate was 78.1, 40.8% for pT_3_N_0_M_0_ groups and pT_1−3_N_1_M_0_ groups, respectively), and thus reinforcing these findings.

LN involvement as well as distant metastasis contributed to the recurrence and progression of disease in RCC patients, resulting in poor survival among advanced RCC patients (11, 21). Pantuck et al. directly compared the survival between 43 (4.8%) patients with pN_0_M_1_ disease and 236 (26%) with pN+M_0_ disease, among the 900 study patients (11). No difference was found for OS between the two groups (*P* = 0.59). MDACC study compared the pT_1−3_N_1_M_0_ disease with pT_1−3_N_0_M_1_ disease in OS and CSS, and found that pT_1−3_N_1_M_0_ disease was not different in both OS (median OS, 2.4y vs. 2.4y, *P* = 0.62) and CSS (median CSS, 2.8y vs. 2.4y, *P* = 0.10) relative to pT_1−3_N_0_M_1_ diseases (12). Meanwhile, in a large cohort analysis of 2,120 patients with RCC from China (13), Shao et al. suggested that T_1−3_N_1_M_0_ disease should be separated from T_3_N_0_M_0_ disease and reclassified as same stage disease with T_4_N_0_M_0_ disease in the AJCC 8th staging system. T_4_N_0_M_0_ disease accounted for almost 79.3% for T_4_M_0_ groups in this study, only 85 T_4_N_1_M_0_ patients were included, while 10,382 patients with M_1_ disease were enrolled, it is not convincing that T_4_N_1_M_0_ groups should be combined with M_1_ disease. However, these studies including this current one indicated that node-positive stage III RCC might need to be separately classified from node-negative stage III disease. In our study, we found the survival of pT_1−3_N_1_M_0_ patients was similar to that of pT_4_N_any_M_0_ patients, and the modified stage that combined pT_1−3_N_1_M_0_ and pT_4_N_any_M_0_ showed improved discriminatory power than 8th AJCC stage. These findings suggest that these two groups might be re-staged together as the same stage.

Pathologic staging of RCC is essential for guiding clinical treatment decision-making process and selecting patients for potential adjuvant therapy (32). High-risk stage II and stage III RCC patients with clear cell histology are considered candidates for adjuvant therapy according to the National Comprehensive Cancer Network (NCCN) guidelines (33). However, two randomized controlled trials compared the adjuvant therapy for non-metastatic RCC patients, and contrary results were found. ASSURE trial showed no differences between sunitinib group and placebo group for stage III/IV patients in disease-free survival (34). Whereas the S-TRAC trial found favorable disease-free survival for sunitinib group in stage III/IV patients (35). In the subset analyses of S-TRAC trial (36), no survival benefit was found in both the low-risk group and high-risk group of T_3_N_0_M_0_ patients by receiving sunitinib. On the contrary, even combined with high-risk group of T_3_N_0_M_0_ patients, T_1−3_N_1_M_0_ patients and T_4_N_any_M_0_ patients were associated with improved survival (HR = 0.74, 95% CI = 0.55–0.99, *P* = 0.04). Given the better survival of T_3_N_0_M_0_ disease in the current study, these controversial findings indicated that T_3_N_0_M_0_ may not significantly benefit from adjuvant therapy similar to T_1−3_N_1_M_0_ and T_4_N_any_M_0_ disease. The poor survival observed for pT_1−3_N_1_M_0_ and pT_4_N_any_M_0_ disease in our study might partly support the necessity of adjuvant therapy. Thus, it might be appropriate to regroup the pT_1−3_N_1_M_0_ disease together with pT_4_N_any_M_0_ disease for better guiding adjuvant therapy.

Over years, the AJCC staging system has aimed to improve its prognostic accuracy by publishing revisions. As a heterogeneous group, locally advanced RCC underwent several staging revisions. The AJCC 7th staging manual reclassified the ipsilateral adrenal invasion into pT_4_ disease for the similar CSS between ipsilateral adrenal invasion and pT_4_ disease (37). Moreover, Anderson et al. (38) showed the similar survival between RCC patients with urinary collecting system invasion (UCSI) and patients with pT_4_ disease, but tumor with UCSI was classified as pT_3a_ in the AJCC 8th staging system. Patel et al. discussed multiple proposed classification systems, and suggested that the understanding and re-evaluation for stage III and IV RCC should be further discussed and validated (39). In the current AJCC 8th cancer staging manual, node-negative disease and node-positive disease in stage III were integrated together into the same stage III disease without further categorization such as stage IIIA and stage IIIB. The higher discriminatory power for our modified stage group in this SEER cohort might also indicate that the current staging system still has room for improve the RCC staging. Nevertheless, the consistency between MDACC results and the current study, and the validation from the large Chinese cohorts and other studies warrants a reexamination of the heterogeneity of the stage III disease defined by the current AJCC 8th cancer staging system in prospective studies.

Our findings must be interpreted within the context of several known limitations. Firstly, as previously described (40), information of invasion beyond capsule, fuhrman nuclear grade, and sarcomatoid features were only applicable after 2010, and not examined in the initial analysis. Nevertheless, subsequent analyses were conducted among cases after 2010, similar results were found among the study groups (Figure S5). Secondly, some important information is not available in SEER database such as performance status (e.g., Eastern Cooperative Oncology Group or Karnofsky performance status), staging methods for patients, comorbidities, and treatment details. These may introduce biases. However, such limitations were also observed in the previous SEER-based analyses (41, 42). Thirdly, there were still some small sample groups in our study, although we could not rule out the biases from unmeasured factors, using PSM methods, well-balanced comparisons might somewhat reduce the biases in our results. It is preferable to obtain more details, however, the remarkable survival difference by nodal status and consistent findings from other cohorts highlighted the future direction for RCC re-staging. We sought to include only the pathologically staged patients and used the pTNM stage throughout the study. Obviously, this may introduce selection bias because we confined our study population to these highly selected patients. However, these highly selected cases represented patients with high-quality medical documentation. It is unlikely the quality of documentation might differ among patients with different stages; therefore, confining our study population to those patients with explicit information should not significantly bias our conclusion. Nevertheless, the present results should be interpreted as preliminary. Further studies, especially large prospective studies, are required to clarify the heterogeneity of the current stage III RCCs classified by AJCC 8th staging system.

In conclusion, the present study suggested that the current AJCC 8th staging system for stage III RCC should be further discussed and validated for the better prediction of prognosis. If validated, patients with pT_1−3_N_1_M_0_ disease might be separated from pT_3_N_0_M_0_ disease (which might be classified as stage IIIA) and classified as stage IIIB together with pT_4_N_any_M_0_ disease. Further researches and independent cohort validations are warranted to address the heterogeneity of the stage III RCCs.

## Data Availability Statement

The datasets generated for this study are available on request to the corresponding author.

## Ethics Statement

Patient consents were not required because the study is a retrospective database research in nature, there was no direct patient contact. Institutional Review Board approval was not required according to our institution policy.

## Author Contributions

ZF: guarantor of the article. ZF and JH: conception/design. JH, QL, PL, SW, YQ, and RZ: collection and/or assembly of data. JH, PL, QL, RZ, SW, YQ, and QS: data analysis and interpretation. JH, QL, PL, SW, RZ, YQ, QS, and ZF: manuscript writing. ZF, JH, PL, SW, QL, RZ, YQ, and QS: final approval of manuscript.

### Conflict of Interest

The authors declare that the research was conducted in the absence of any commercial or financial relationships that could be construed as a potential conflict of interest.

## References

[1] GLOBOCAN 2018: renal cell carcinoma: estimated cancer incidence, mortality and prevalence worldwide in 2018 International Agency for Research on Cancer: World Health Organization. (2018). Available online at: http://gco.iarc.fr/today/fact-sheets-populations (accessed December 23, 2018).

[2] Capitanio U Suardi N Matloob R Roscigno M Abdollah F Di Trapani E. Extent of lymph node dissection at nephrectomy affects cancer-specific survival and metastatic progression in specific sub-categories of patients with renal cell carcinoma (RCC). BJU Int. (2014) 114:210–5. 10.1111/bju.12508 24854206

[3] Tilki D Chandrasekar T Capitanio U Ciancio G Daneshmand S Gontero P. Impact of lymph node dissection at the time of radical nephrectomy with tumor thrombectomy on oncological outcomes: results from the international renal cell carcinoma-venous thrombus consortium (IRCC-VTC). Urol Oncol. (2018) 36:79e11–17. 10.1016/j.urolonc.2017.10.008 29129353 PMC8404533

[4] Capitanio U Jeldres C Patard JJ Perrotte P Zini L de La Taille A. Stage-specific effect of nodal metastases on survival in patients with non-metastatic renal cell carcinoma. BJU Int. (2009) 103:33–7. 10.1111/j.1464-410X.2008.08014.x 18990161

[5] Terrone C Cracco C Porpiglia F Bollito E Scoffone C Poggio M. Reassessing the current TNM lymph node staging for renal cell carcinoma. Eur Urol. (2006) 49:324–31. 10.1016/j.eururo.2005.11.014 16386352

[6] Lughezzani G Capitanio U Jeldres C Isbarn H Shariat SF Arjane P. Prognostic significance of lymph node invasion in patients with metastatic renal cell carcinoma: a population-based perspective. Cancer. (2009) 115:5680–7. 10.1002/cncr.24682 19824083

[7] Blute ML Leibovich BC Cheville JC Lohse CM Zincke H. A protocol for performing extended lymph node dissection using primary tumor pathological features for patients treated with radical nephrectomy for clear cell renal cell carcinoma. J Urol. (2004) 172:465–9. 10.1097/01.ju.0000129815.91927.85 15247704

[8] Amin M Edge S Greene F AJCC Cancer Staging Manual. 8th ed New York, NY: Springer (2017).

[9] Canfield SE Kamat AM Sánchez-Ortiz RF Detry M Swanson DA Wood CG. Renal cell carcinoma with nodal metastases in the absence of distant metastatic disease (clinical stage TxN1-2M0): the impact of aggressive surgical resection on patient outcome. J Urol. (2006) 175:864–9. 10.1016/S0022-5347(05)00334-4 16469567

[10] Trinh QD Schmitges J Bianchi M Sun M Shariat SF Sammon J. Node-positive renal cell carcinoma in the absence of distant metastases: predictors of cancer-specific mortality in a population-based cohort. BJU Int. (2012) 110:E21–7. 10.1111/j.1464-410X.2011.10701.x 22044638

[11] Pantuck AJ Zisman A Dorey F Chao DH Han KR Said J. Renal cell carcinoma with retroperitoneal lymph nodes: role of lymph node dissection. J Urol. (2003) 169:2076–83. 10.1097/01.ju.0000066130.27119.1c 12771723

[12] Yu KJ Keskin SK Meissner MA Petros FG Wang X Borregales LD. Renal cell carcinoma and pathologic nodal disease: implications for American Joint Committee on cancer staging. Cancer. (2018) 124:4023–31. 10.1002/cncr.31661 30276798

[13] Shao N Wang HK Zhu Y Ye DW. Modification of American Joint Committee on cancer prognostic groups for renal cell carcinoma. Cancer Med. (2018) 7:5431–8. 10.1002/cam4.1790 30306741 PMC6247054

[14] NCI Overview of the SEER program. (2018). Available online at: https://seer.cancer.gov/about/overview.html (accessed December 23, 2018).

[15] Surgery Codes of Kidney. (2018). Available online at: https://seer.cancer.gov/manuals/2018/AppendixC/Surgery_Codes_Kidney_2018.pdf (accessed December 23, 2018).

[16] NCI Collaborative Stage Site-Specific Factors (CS SSF). (2018). Available online at: https://seer.cancer.gov/seerstat/databases/ssf/ (accessed December 23, 2018).

[17] Rosenbaum PR Rubin DB The central role of the propensity score in observational studies for causal effects. Biometrika. (1983) 70:41–55. 10.1093/biomet/70.1.41

[18] Hollingsworth JM Miller DC Daignault S Hollenbeck BK. Five-year survival after surgical treatment for kidney cancer: a population-based competing risk analysis. Cancer. (2007) 109:1763–8. 10.1002/cncr.22600 17351954

[19] Verhoest G Veillard D Guille F De La Taille A Salomon L Abbou CC. Relationship between age at diagnosis and clinicopathologic features of renal cell carcinoma. Eur Urol. (2007) 51:1298–304. 10.1016/j.eururo.2006.11.056 17174023

[20] Minervini A Lilas L Minervini R Selli C. Prognostic value of nuclear grading in patients with intracapsular (pT1-pT2) renal cell carcinoma. Long-term analysis in 213 patients. Cancer. (2002) 94:2590–5. 10.1002/cncr.10510 12173325

[21] Sun M Bianchi M Hansen J Abdollah F Trinh QD Lughezzani G. Nodal involvement at nephrectomy is associated with worse survival: a stage-for-stage and grade-for-grade analysis. Int J Urol. (2013) 20:372–80. 10.1111/j.1442-2042.2012.03170.x 23039208

[22] RMS: Regression Modeling Strategies. R package version 3.5.1. Available online at: https://CRAN.R-project.org/package=rms (accessed December 23, 2018).

[23] Wang Y Li J Xia Y Gong R Wang K Yan Z. Prognostic nomogram for intrahepatic cholangiocarcinoma after partial hepatectomy. J Clin Oncol. (2013) 31:1188–95. 10.1200/JCO.2012.41.5984 23358969

[24] Schroeder M Culhane A Quackenbush J Haibe-Kains B. survcomp: an R/Bioconductor package for performance assessment and comparison of survival models. Bioinformatics. (2011) 27:3206–8. 10.1093/bioinformatics/btr511 21903630 PMC3208391

[25] Pu N Li J Xu Y Lee W Fang Y Han X. Comparison of prognostic prediction between nomogram based on lymph node ratio and AJCC 8th staging system for patients with resected pancreatic head carcinoma: a SEER analysis. Cancer Manag Res. (2018) 10:227–38. 10.2147/CMAR.S157940 29440932 PMC5804271

[26] Blom JH van Poppel H Marechal JM Jacqmin D Schroder FH de Prijck L. Radical nephrectomy with and without lymph-node dissection: final results of European organization for research and treatment of cancer (EORTC) randomized phase 3 trial 30881. Eur Urol. (2009) 55:28–34. 10.1016/j.eururo.2008.09.052 18848382

[27] Pizzocaro G Piva L. Pros and cons of retroperitoneal lymphadenectomy in operable renal cell carcinoma. Eur Urol. (1990) 18(Suppl.2):22–3. 10.1159/000463953 2226599

[28] Giberti C Oneto F Martorana G Rovida S Carmignani G. Radical nephrectomy for renal cell carcinoma: long-term results and prognostic factors on a series of 328 cases. Eur Urol. (1997) 31:40–8. 10.1159/000474416 9032533

[29] Antonelli A Cozzoli A Zani D Zanotelli T Nicolai M Cunico SC. The follow-up management of non-metastatic renal cell carcinoma: definition of a surveillance protocol. BJU Int. (2007) 99:296–300. 10.1111/j.1464-410X.2006.06616.x 17326263

[30] Lam JS Shvarts O Pantuck AJ. Changing concepts in the surgical management of renal cell carcinoma. Eur Urol. (2004) 45:692–705. 10.1016/j.eururo.2004.02.002 15149740

[31] Srivastava A Rivera-Nunez Z Kim S Sterling J Farber N Radadia K mp14-20 Impact of pathologic node positive renal cell carcinoma on survival in patients without Metastasis: evidence in support of expanding the definition of stage IV kidney cancer. J Urol. (2019) 201(Supp.l4):e195 10.1097/01.JU.0000555316.15591.47 32329899

[32] Miller LA Stemkowski S Saverno K Lane DC Tao Z Hackshaw MD. Patterns of care in patients with metastatic renal cell carcinoma among a U.S. payer population with commercial or medicare advantage membership. J Manag Care Spec Pharm. (2016) 22:219–26. 10.18553/jmcp.2016.22.3.219 27003551 PMC10397977

[33] Kidney cancer, Version 2 NCCN clinical practice guidelines in oncology. National Comprehensive Cancer Network. (2018). Available online at: https://www.nccn.org/professionals/physician_gls/default.aspx (accessed January 21, 2019).

[34] Haas NB Manola J Uzzo RG Flaherty KT Wood CG Kane C. Adjuvant sunitinib or sorafenib for high-risk, non-metastatic renal-cell carcinoma (ECOG-ACRIN E2805): a double-blind, placebo-controlled, randomised, phase 3 trial. Lancet. (2016) 387:2008–16. 10.1016/S0140-6736(16)00559-6 26969090 PMC4878938

[35] Ravaud A Motzer RJ Pandha HS George DJ Pantuck AJ Patel A. Adjuvant sunitinib in high-risk renal-cell carcinoma after nephrectomy. N Engl J Med. (2016) 375:2246–54. 10.1056/NEJMoa1611406 27718781

[36] Motzer RJ Ravaud A Patard JJ Pandha HS George DJ Patel A. Adjuvant sunitinib for high-risk renal cell carcinoma after nephrectomy: subgroup analyses and updated overall survival results. Eur Urol. (2018) 73:62–8. 10.1016/j.eururo.2017.09.008 28967554 PMC6684251

[37] Edge S Byrd D Compton C Fritz A Greene F Trotti A AJCC Cancer Staging Manual. 7th ed New York, NY: Springer (2010).

[38] Anderson CB Clark PE Morgan TM Stratton KL Herrell SD Davis R. Urinary collecting system invasion is a predictor for overall and disease-specific survival in locally invasive renal cell carcinoma. Urology. (2011) 78:99–104. 10.1016/j.urology.2011.02.039 21550647

[39] Patel HV Srivastava A Shinder B Sadimin E Singer EA. Strengthening the foundation of kidney cancer treatment and research: revising the AJCC staging system. Ann Transl Med. (2019) 7(Suppl. 1):S33–37 10.21037/atm.2019.02.19 31032312 PMC6462582

[40] Altekruse SF Dickie L Wu XC Hsieh MC Wu M Lee R. Clinical and prognostic factors for renal parenchymal, pelvis, and ureter cancers in SEER registries: collaborative stage data collection system, version 2. Cancer. (2014) 120(Suppl.23):3826–35. 10.1002/cncr.29051 25412394 PMC4612347

[41] Joslyn SA Sirintrapun SJ Konety BR. Impact of lymphadenectomy and nodal burden in renal cell carcinoma: retrospective analysis of the national surveillance, epidemiology, and end results database. Urology. (2005) 65:675–80. 10.1016/j.urology.2004.10.068 15833507

[42] Daskivich TJ Tan HJ Litwin MS Hu JC. Life expectancy and variation in treatment for early stage kidney cancer. J Urol. (2016) 196:672–7. 10.1016/j.juro.2016.03.133 27012644

