# Supplementary material for: Reassessment of American Joint Committee on Cancer Staging for Stage III Renal Cell Carcinoma With Nodal Involvement: Propensity Score Matched Analyses of a Large Population-Based Study
Source: Front Oncol. 2020 Mar 19;10:365. doi: 10.3389/fonc.2020.00365 (PMC7096477; doi:10.3389/fonc.2020.00365)
Supplement: Supplementary file 1 [file Data_Sheet_1.pdf]

Table S1. Baseline characteristics of $pT_4N_{any}M_0$ and $pT_{any}N_{any}M_1$ groups before and after matching

Characteristic	Before Matching			After Matching*		
	Stage IV-M0 $pT_4N_{any}M_0$ (N=105, %)	Stage IV-M1 $pT_{any}N_{any}M_1$ (N=1007, %)	*P* †	Stage IV-M0 $pT_4N_{any}M_0$ (N=105, %)	Stage IV-M1 $pT_{any}N_{any}M_1$ (N=105, %)	*P* †
Age, mean (95%CI), y	59.7 (57.7-61.6)	58.2 (57.6-58.9)	0.18	59.7 (57.8-61.7)	58.9 (56.7-61.0)	0.58
Sex			0.091			0.98
Male	65 (61.9)	704 (69.9)		65 (61.9)	65 (61.9)	
Female	40 (38.1)	303 (30.1)		40 (38.1)	40 (38.1)	
Race			0.41			0.094
White	88 (83.8)	873 (86.7)		88 (83.8)	96 (91.4)	
Nonwhite	17 (16.2)	134 (13.3)		17 (16.2)	9 (8.6)	
Grade			0.56			0.99
Well/Moderately	13 (12.4)	164 (16.3)		13 (12.4)	13 (12.4)	
Poor	53 (50.5)	441 (43.8)		53 (50.5)	53 (50.5)	
Undifferentiated	32 (30.5)	327 (32.5)		32 (30.5)	32 (30.5)	
Unknown	7 (6.7)	75 (7.4)		7 (6.7)	7 (6.7)	
Tumor site			0.79			0.49
Left	60 (57.1)	561 (55.7)		60 (57.1)	55 (52.4)	
Right	45 (42.9)	446 (44.3)		45 (42.9)	50 (47.6)	
Histology			0.011			0.99
Clear cell	84 (80.0)	899 (89.3)		84 (80.0)	84 (80.0)	
Chrompohobe	6 (5.7)	22 (2.2)		6 (5.7)	6 (5.7)	
Papillary	15 (14.3)	86 (8.5)		15 (14.3)	15 (14.3)	
T stage			NA			NA
T1	0 (0.0)	66 (6.6)		0 (0.0)	8 (7.7)	
T2	0 (0.0)	160 (15.9)		0 (0.0)	10 (9.6)	
T3a	0 (0.0)	382 (37.9)		0 (0.0)	43 ((41.0)	
T3b	0 (0.0)	247 (245)		0 (0.0)	26 (24.8)	
T3c	0 (0.0)	21 (2.1)		0 (0.0)	1 (1.0)	
T3NOS	0 (0.0)	18 (1.8)		0 (0.0)	1 (1.0)	
T4	105 (100.0)	113 (11.2)		105 (100.0)	16 (15.2)	
N stage			0.001			0.011
N0	88 (83.8)	585 (58.1)		88 (83.8)	47 (44.8)	
N1	17 (16.2)	422 (41.7)		17 (16.2)	58 (55.2)	
Invasion beyond capsule‡			NA			NA
No	12 (17.1)	200 (35.3)		12 (17.1)	17 (27.9)	
Yes	56 (80.0)	321 (56.6)		56 (80.0)	38 (62.3)	
Unknown	2 (2.9)	46 (8.1)		2 (2.9)	6 (9.8)	
Fuhrman nuclear grade‡			NA			NA
Grade 1/2	10 (14.3)	87 (16.3)		10 (14.3)	9 (14.8)	
Grade 3	33 (47.1)	231 (40.7)		33 (47.1)	20 (32.8)	
Grade 4	25 (35.7)	214 (37.7)		25 (35.7)	23 (37.7)	
Unknown	2 (2.9)	35 (6.2)		2 (2.9)	9 (14.8)	

Table S1. Continued

Sarcomatoid features‡			NA			NA
Absence	55 (78.6)	459 (81.0)		55 (78.6)	48 (78.7)	
Presence	14 (20.0)	83 (14.6)		14 (20.0)	8 (13.1)	
Unknown	1 (1.4)	25 (4.4)		1 (1.4)	5 (8.2)	
LNs examined, mean (95%CI)	8 (6-10)	8 (7-9)	0.99	8 (6-10)	8 (6-11)	0.99
LNs positive, mean (95%CI)	1 (0-2)	2 (1-3)	0.18	1 (0-2)	2 (1-2)	0.1

Abbreviation: LN: lymph node; CI: Confidence interval; NA: Not Applicable

*Adjusted for group, age, grade, and histology.

†Derived from χ2-test for categorical variables, general linear models for continuous variables.

‡ Invasion beyond capsule, fuhrman nuclear grade, and sarcomatoid features were only applicable for 2010+ cases, and not enter into the χ2-test, thus the *P* value was not applicable.

Table S2. Univariate and multivariate analyses of overall survival and cause-specific survival to $pT_3N_0M_0$ and $pT_{1-3}N_1M_0$ groups after matching

Characteristic	Overall Survival						Cancer-specific Survival					
	Univariate			Multivariate†			Univariate			Multivariate†		
	HR	95% CI	*P*	HR	95% CI	*P*	HR	95% CI	*P*	HR	95% CI	*P*
Age	1.02	1.01-1.03	0.001	1,02	1.01-1.03	0.001	1.01	1.00-1.02	0.046	1.01	1.00-1.02	0.040
Sex												
Male (ref.)	1.0	1.0		/	/	/	1.0	1.0		/	/	/
Female	0.95	0.74-1.2	0.69	/	/	/	1.0	0.78-1..3.	0.89	/	/	/
Race												
White (ref.)	1.0	1.0		/	/	/	1.0	1.0		/	/	/
Nonwhite	1.1	0.83-1.5	0.48	/	/	/	1.1	0.80-1.5	0.56	/	/	/
Grade												
Well/Moderately (ref.)	1.0	1.0		1.0	1.0		1.0	1.0		1.0	1.0	
Poor	1.3	0.92-1.8	0.14	1.3	0.96-1.8	0.083	1.3	0.94-1.9	0.10	1.4	0.98-2.0	0.069
Undifferentiated	1.9	1.4-2.7	0.001	2.0	1.4-2.8	<0.001	2.0	1.4-2.9	<0.001	2.1	1.5-3.0	<0.001
Unknown	1.1	0.68-1.9	0.62	1.2	0.73-2.1	0.43	1.3	0.78-2.3	0.30	1.4	0.81-2.4	0.24
Tumor site												
Left (ref.)	1.0	1.0		/	/	/	1.0	1.0		1.0	1.0	
Right	1.2	0.98-1.5	0.078	/	/	/	1.3	1.0-1.6	0.051	1.3	1.0-1.6	0.049
Histology												
Clear cell (ref.)	1.0	1.0		1.0	1.0		1.0	1.0		1.0	1.0	
Chrompohobe	0.30	0.13-0.68	0.004	0.30	0.13-0..69	0.004	0.28	0.12-0.68	0.005	0.29	0.12-0.70	0.006
Papillary	1.2	0.89-1.6	0.24	1.2	0.92-1.7	0.17	1.2	0.88-1.6	0.24	1.3	0.92-1.7	0.16
Invasion beyond capsule‡												
No (ref.)	1.0	1.0		/	/	/	1.0	1.0		/	/	/
Yes	1.1	0.75-1.5	0.76	/	/	/	0.80	0.44-1.5	0.47	/	/	/
Unknown	1.3	0.75-2.3	0.33	/	/	/	0.79	0.43-1.4	0.46	/	/	/
Fuhrman nuclear grade‡												
Grade 1/2 (ref.)	1.0	1.0		1.0	1.0		1.0	1.0		1.0	1.0	
Grade 3	1.6	0.92-2.8	0.096	1.4	0.80-2.5	0.23	1.8	0.96-3.3	0.067	1.5	0.83-2.9	0.17

Table S2. Continued.

Grade 4	3.5	2.0-6.0	<0.001	2.5	1.4-4.4	0.001	3.7	2.0-6.8	<0.001	2.6	1.4-4.8	0.003
Unknown	2.0	0.98-4.2	0.058	1.4	0.63-2.9	0.44	2.5	1.2-5.4	0.020	1.6	0.70-3.5	0.28
Sarcomatoid features‡												
Absence (ref.)	1.0	1.0		1.0	1.0		1.0	1.0		1.0	1.0	
Presence	2.6	1.7-3.9	<0.001	1.7	1.1-2.7	0.016	2.7	1.8-4.2	<0.001	1.8	1.2-2.9	0.009
Unknown	2.1	0.93-4.8	0.075	1.9	0.75-4.6	0.18	2.4	1.1-5.6	0.035	2.0	0.77-5.1	0.16
LNs examined	1.00	0.99-1.01	0.74	/	/	/	1.00	0.99-1.01	0.63	/	/	/
LNs positive	1.01	1.00-1.01	0.18	/	/	/	1.01	1.00-1.01	0.21	/	/	/
Group												
$pT_3N_0M_0$ (ref.)	1.0	1.0		1.0	1.0		1.0	1.0		1.0	1.0	
$pT_{1\text{-}3}N_1M_0$	2.3	1.8-2.9	<0.001	2.5	1.9-3.1	<0.001	2.4	1.8-3.1	<0.001	2.5	2.0-3.3	<0.001

Abbreviation: HR: hazards ratio; CI: confidence intervals; LN: lymph node; ref.: referent

*Adjusted for group, age, grade, and histology

†Adjusted for age, group, grade, and histology for both overall survival and cause-specific survival.

‡ Invasion beyond capsule, fuhrman nuclear grade, and sarcomatoid features were only applicable for 2010+ cases, and adjusted for age, group, histology for 2010+ cases

Figure S1. Propensity score distribution* for stage III-N0 and III-N1 groups (A), stage III-N1 and IV-M0 groups (B), stage IV-M0 and IV-M1 groups (C), and stage III-N1 and IV-M1 groups (D).

*Dot-plot demonstrate the distribution of propensity scores for related groups in renal cell carcinoma patients. Stratified into matched and unmatched categories.

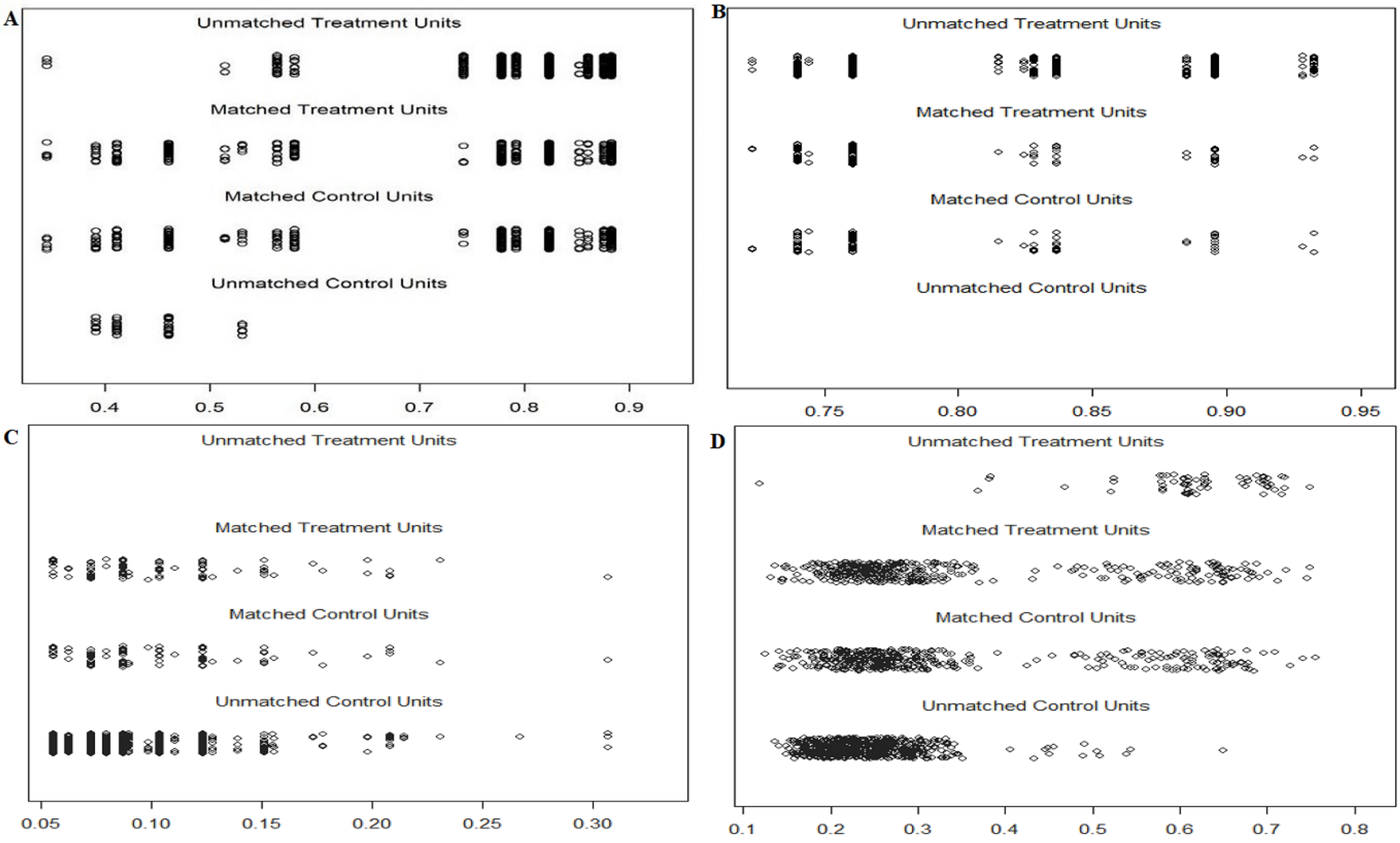

Figure S2. Kaplan-Meire estimation of overall survival curves (before matching: A; After matching: C) and cancer-specific survival curves (before matching: B; After matching: D) relative to each histology groups.

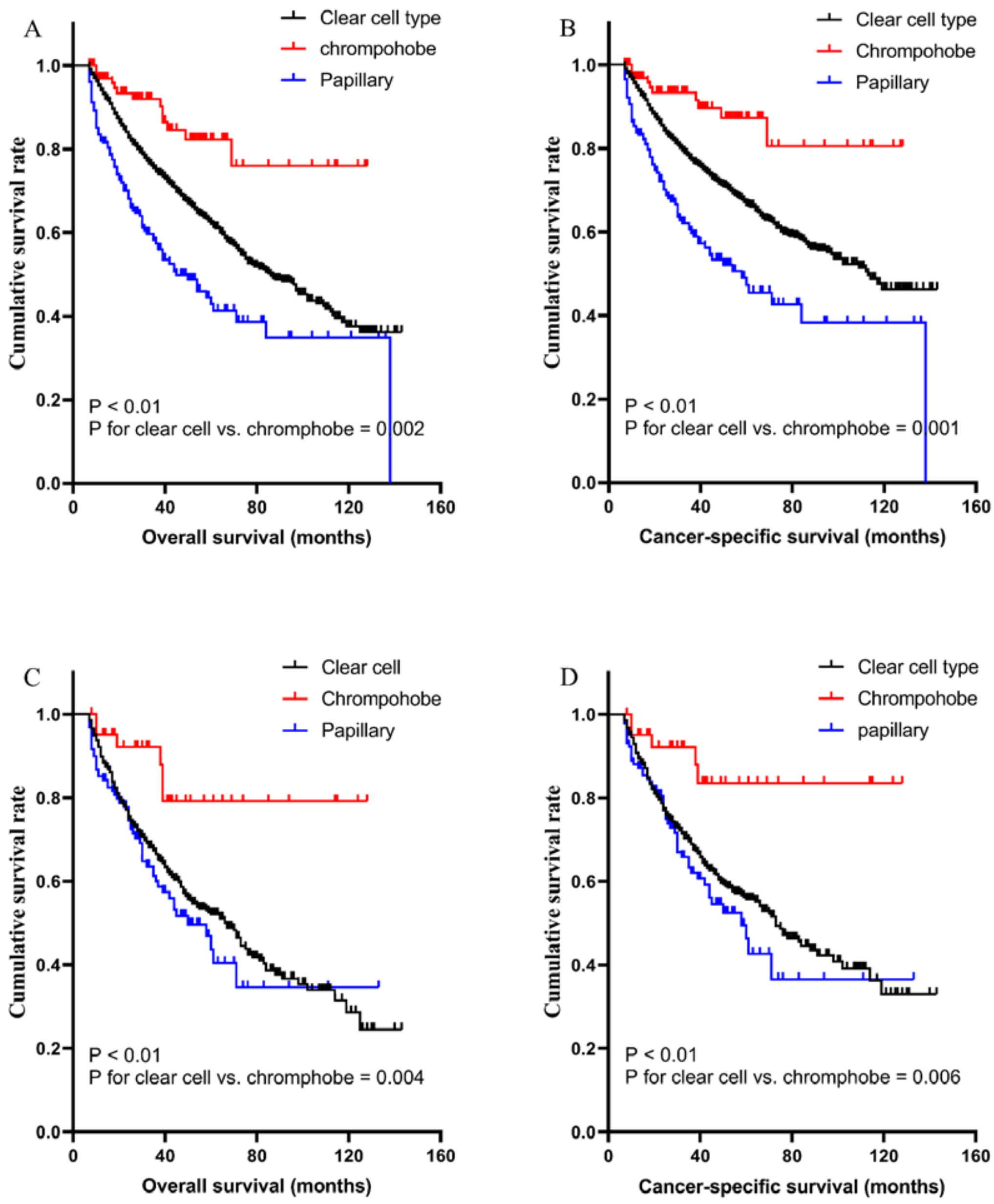

Figure S3. Kaplan-Meire estimation of overall survival curves (A) and cancer-specific survival curves (B) relative to stage III-N1 and stage IV-M0 groups after matching.

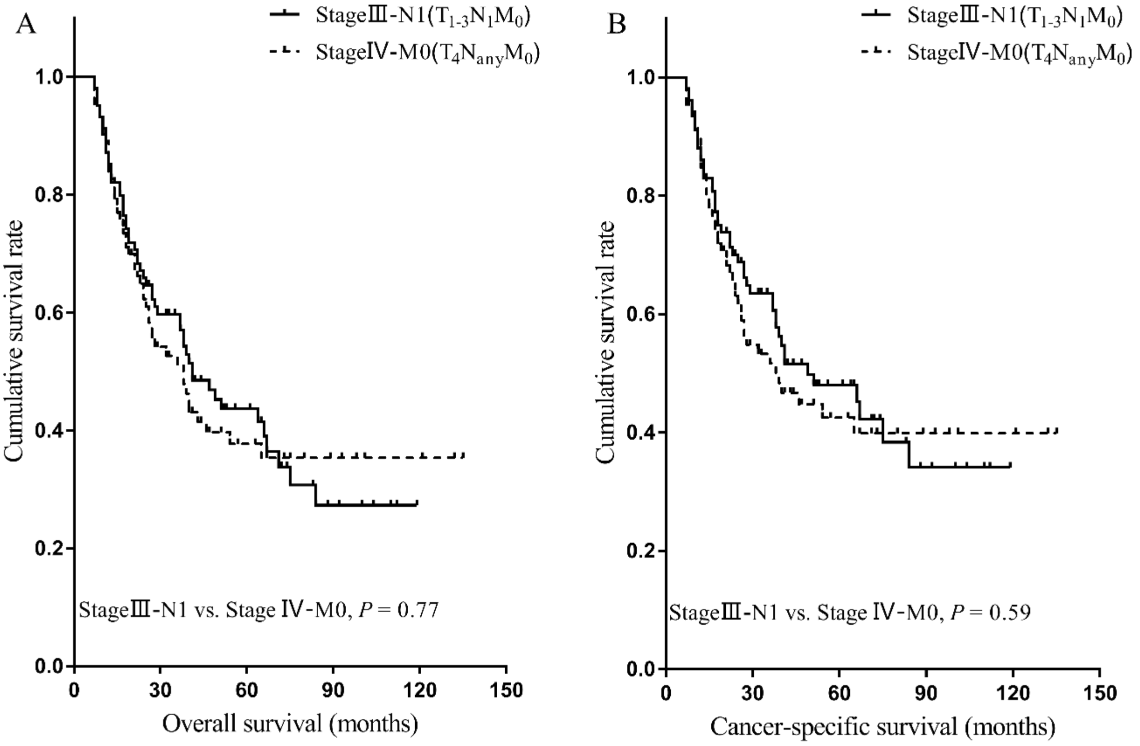

Number at risk

StageⅢ-N1	105	49	22	8	1
StageⅣ-M0	105	38	19	9	4

Number at risk

StageⅢ-N1	105	49	21	8	1
StageⅣ-M0	105	38	19	9	4

Figure S4. Kaplan-Meire estimation of overall survival curves (before matching: A; After matching: C) and cancer-specific survival curves (before matching: B; After matching: D) relative to each groups.

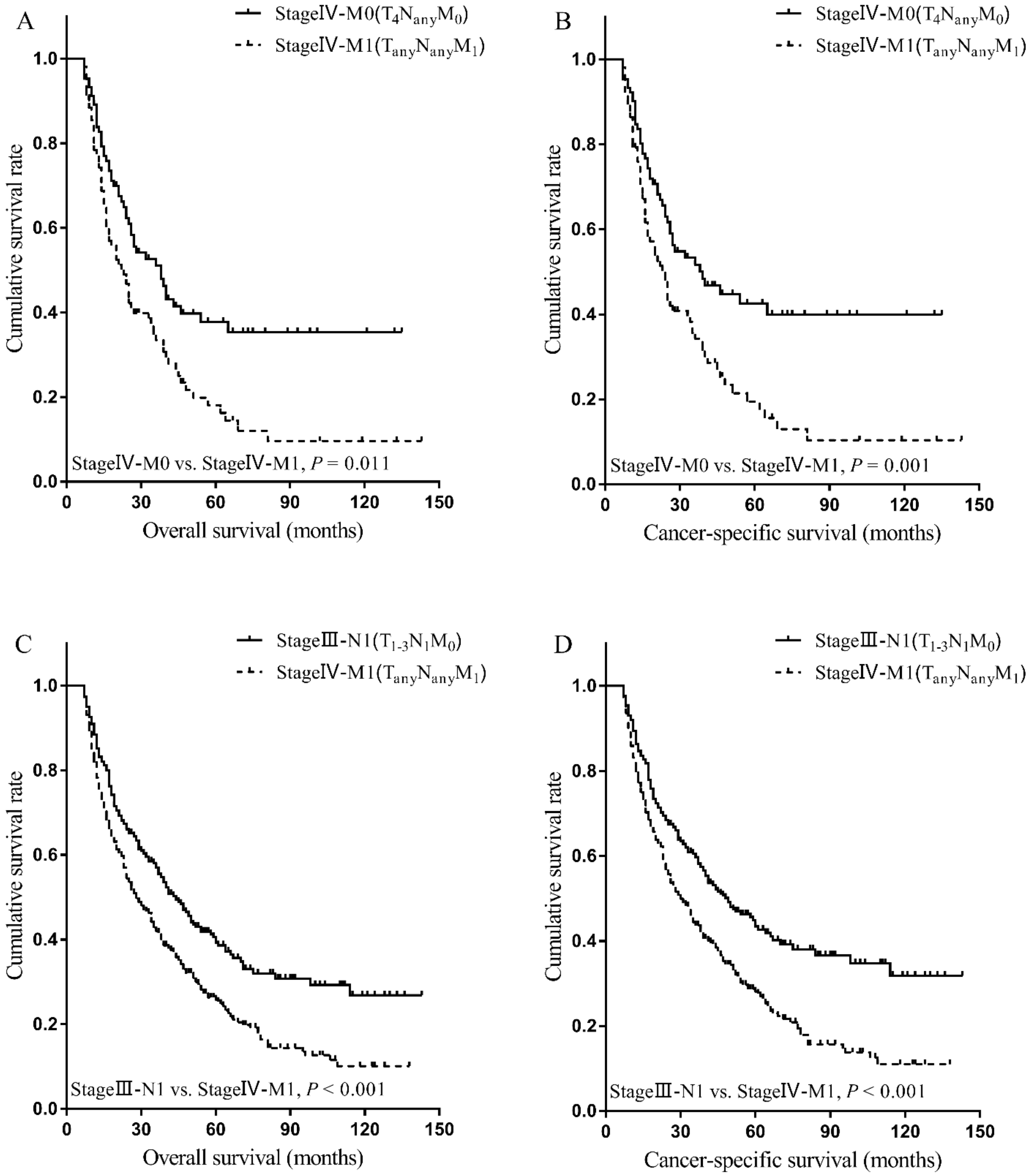

Figure S5. Kaplan-Meire estimation of overall survival curves (before matching*: A; After matching*: C) and cancer-specific survival curves (before matching*: B; After matching*: D) relative to each group for cases diagnosed after 2010.

.

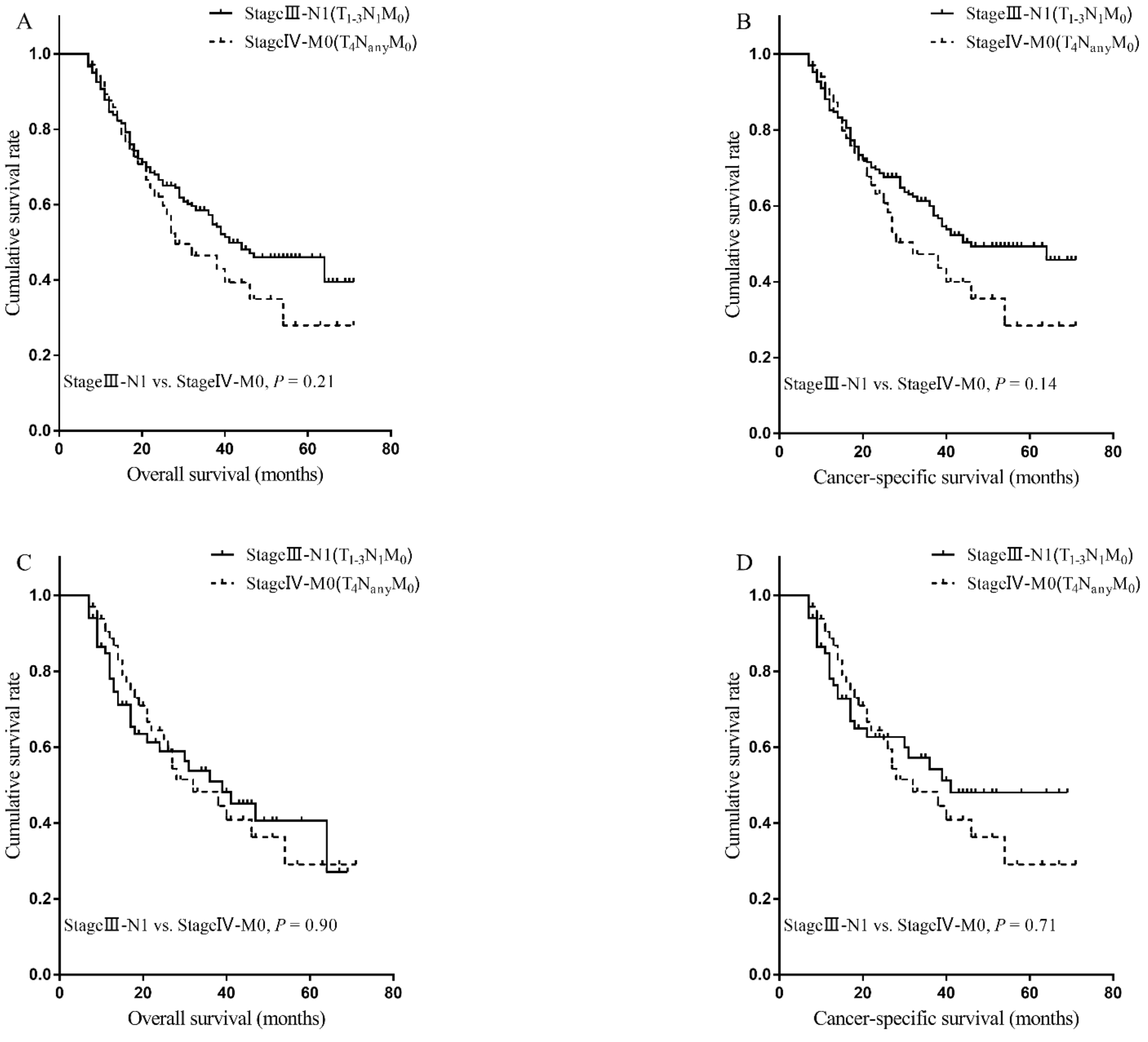

*For cases diagnosed after 2010, age, group, grade, histology, and sarcomatoid features were matched for both overall survival and cause-specific survival

Figure S6. Prognostic nomogram, calibration curves of overall survival (OS) for modified stage of study patients. Notes: (A) The nomogram predicts 1-, 3-, and 5-year OS in patients with renal cell carcinoma. The calibration curves predicts OS at 1 year (B), 3 year (C), and 5 year (D) in the primary cohort and at 1 year (E), 3 year (F), and 5 year (G) in the validation cohort.

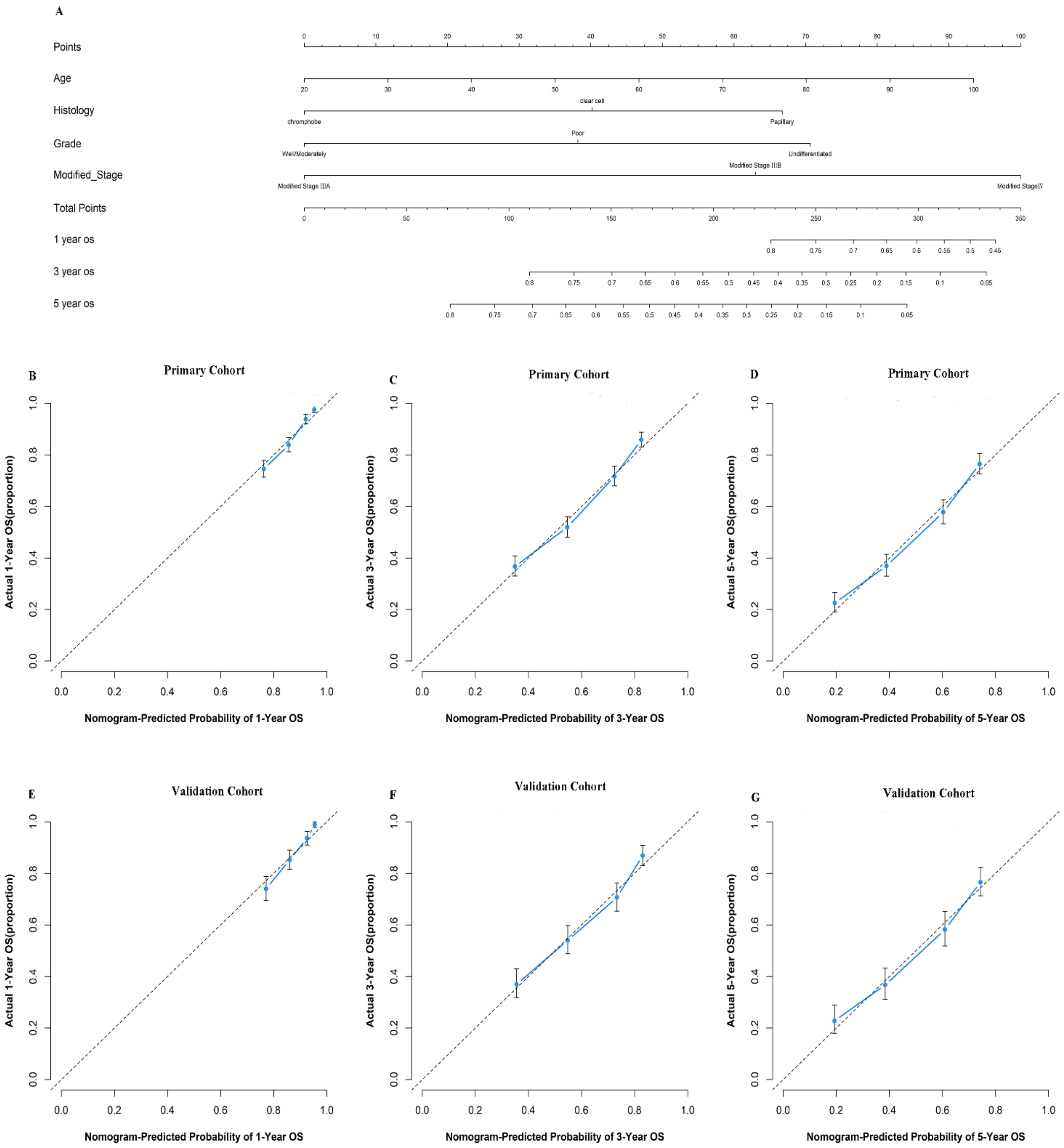

Code for generating the nomogram with R

```
library(rms)
library(Hmisc)
library(survival)
library(survcomp)
```

Generate Cox Proportional Hazards Model

```
Cox_model1 <- cph(Surv(Time,Status) ~ AJCC_Stage+…, x=TRUE, y=TRUE, surv=TRUE, time.inc = 12 or 36 or 60);
Cox_model2 <- cph(Surv(Time,Status) ~ Modified_Stage+…, x=TRUE, y=TRUE, surv=TRUE, time.inc = 12 or 36 or 60);
```

Generate npmogram

```
survival1 <- Survival(Cox_model 1 or 2)
survival11 <- function(x)survival1(12,x) #1 year
survival12 <- function(x)survival1(36,x) #3 year
survival13 <- function(x)survival1(60,x) #5 year
nom <- nomogram(f1, fun=list(survival11, survival12, survival13), fun.at=c(0.05, seq(0.1, 0.75, by=0.05), 0.80), lp=F, funlabel=c('1 year survival', '3 year survival', '5 year survival'))
plot(nom, cex.axis = 0.8,cex.lab=0.8)
```

For inner validation group

```
data <- read.csv("file location") #change to your dataset
data_v <- sample(nrow(your file), sample number, replace=FALSE)
data_validation <- data [data _v,]
data _validation <- read.csv("file location")
```

compute C-index and compare C-index

```
validate(Cox_model 1 or 2, method="boot", B=1000, dxy=T)
x1 <- predict(Cox_model1)
x2 <- predict(Cox_model2)
c1 <- concordance.index(x1,surv.time=Time, surv.event=Status, method="noether")
c2 <- concordance.index(x2,surv.time=Time, surv.event=Status, method="noether")
print(c(c1[1],c1$lower,c1$upper))
print(c(c2[1],c2$lower,c2$upper))
cindex.comp(c1,c2)
```

For calibration curve

```
calm <- calibrate(Cox_model, cmethod="KM", method="boot", u=12 or 36 or 60, m=600, B=1000)
opar <- par(no.readonly=T)
```

```
par(mar=c(8,5,3,2),cex=1.0)
plot(calm, lwd=1, lty=1, bty="n", subtitles = " ", font.lab = "2", errbar.col=c(rgb(0,0,0,maxColorValue=255)),
xlim=c(0,1), ylim=c(0,1), xlab="Nomogram-Predicted Probability", ylab="Actual survival",
col=c(rgb(192,98,83,maxColorValue=255)))
lines(calm[,c("mean.predicted","KM")],type="b",lwd=2,col=c(rgb(30,144,255,maxColorValue=255)),pch=16)
abline(0,1,lty=2,lwd=1,col=c(rgb(0,0,0,maxColorValue=255)))
par(opar)
```
